# High yield accelerated reactions in nonvolatile microthin films: chemical derivatization for analysis of single-cell intracellular fluid†
†Dedicated to Keith R. Jennings, a pioneer in mass spectrometry, on his 85^th^ birthday.
‡
‡Electronic supplementary information (ESI) available. See DOI: 10.1039/c8sc03382j


**DOI:** 10.1039/c8sc03382j

**Published:** 2018-08-21

**Authors:** Zhenwei Wei, Xiaochao Zhang, Jinyu Wang, Sichun Zhang, Xinrong Zhang, R. Graham Cooks

**Affiliations:** a Department of Chemistry , Purdue University , West Lafayette , IN 47907 , USA . Email: cooks@purdue.edu; b Department of Chemistry , Tsinghua University , Beijing Key Lab Microanalytical Methods and Instruments , Beijing 100084 , P. R. China . Email: xrzhang@tsinghua.edu.cn

## Abstract

The identification of trace components from biological media can require derivatization under mild conditions for successful analysis by mass spectrometry (MS). When aqueous droplets (*ca.* 500 nL) containing a sugar and an amine as reagents are allowed to evaporate they may form long-lasting microthin films in which derivatization reactions can occur fast relative to reaction rates in bulk solution. Evidence is presented that these reactions are heterogeneous in nature and comparisons are made with reactions in pastes and in neat reagent mixtures. Moreover, these thin film reactors can be made stable for the long periods of time that may be necessary to give high product yields. The situation is typified by imine formation from reducing sugars which have reaction times of much more than 1 hour provided that small concentrations (*e.g.* 20 ppm) of nonvolatile solvents are included. After evaporation of almost all the water, the reaction occurs at an approximately constant rate for the first hour. The rate is two orders of magnitude faster than the reaction in the corresponding homogeneous saturated bulk solution. Conversion of the reagent to the Schiff base product is 67% to 96% efficient in these long-lasting thin films, in sharp contrast to the corresponding derivatization efficiencies in the bulk of less than 1%. This method was used to chemically derivatize and thus to identify, using tandem mass spectrometry, 29 reducing sugars in *ca.* 1 nL of intracellular fluid from a single onion epidermis cell. A formal description of the kinetics of reversible and irreversible second order reactions in thin films is provided. The effects of thermodynamic and kinetic factors are separated and the measured apparent acceleration factor is shown to represent the ratio of intrinsic rate constants for the microthin film reactor relative to the bulk reaction.

## Abstract 2

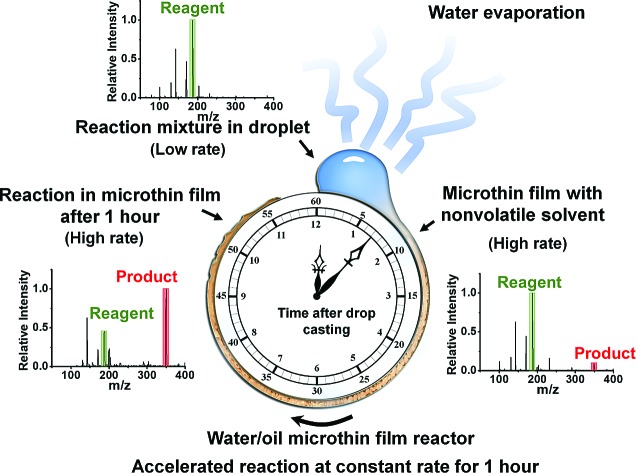
The identification of trace components from an individual cell can require derivatization under mild conditions for successful analysis by mass spectrometry (MS).

## Introduction

Chemical derivatization is an important strategy to increase analytical performance,^1^–^5^ especially analytical sensitivity and selectivity,^6^,^7^ and it has been widely used for profiling metabolites,^8^,^9^ quantifying bioactive small molecules and identifying natural products.^10^–^12^ Recently, new demands have been placed on chemical derivatization in the analysis of small amounts of compounds in microvolume samples derived from individual cells.^13^ Especially significant on very small scales is the fact that derivatization can lead to loss of analytes due to unsatisfactory reaction yields. The problems in utilizing chemical derivatization for small volume assays, especially in single-cell analysis, can be alleviated by addressing two questions: (1) can derivatization reactions be accelerated? (2) Can the yields of derivatization reactions be improved?

In the recent past, several novel routes to access fast reactions and unique reactivity have been reported. For example, in “on water” chemistry, organic reactions take place in an aqueous/organic emulsion. These reactions exhibit reaction rate acceleration and unique reactivity compared to bulk reactions conducted in solvents or under neat conditions.^14^,^15^ Very recently, prebiotic mimic chemistry has established that phosphorylation of nucleosides can proceed rapidly and in high yield in an aqueous paste of highly concentrated reactants with minimum solvent.^16^ In these two cases, reaction acceleration and unique reactivity take place in a solvent-poor system where high concentrations and limited solvation drive the reaction. Another somewhat similar case uses flow chemistry in an ionic liquid droplet reactor to catalyze reactions and achieve higher rates and conversion ratios with a special reactive surface.^17^,^18^ Compared to homogeneous bulk phase reactions, these emerging studies using highly reactive interfaces lead to high reaction rates and favorable product yields. In addition to these cases, reactions are also known to be accelerated by one to five orders of magnitude^19^ relative to those in bulk when they take place in small confined volumes such as in electrosprayed microdroplets,^20^–^25^ levitated neutral droplets^26^–^28^ or thin films.^20^,^28^–^30^ In each of these systems, the reaction mixture undergoes solvent evaporation to generate large surface to volume ratio compartments where reactants are concentrated and rate constants may be increased by interfacial effects.^31^

When manipulating single cell samples for chemical analysis dilution is usually involved and this decreases the already low concentrations of target molecules. Pre-concentration of compounds derived from single cells may be necessary to achieve satisfactory derivatization yields. Reactions in microdroplets or thin films are especially attractive for such cases because solvent evaporation will not only bring reactant concentration into a useful range but the increased surface-to-volume ratio will also increase rate constants and reaction yields. As accelerated reaction rates and satisfactory yields follow upon solvent evaporation, the issue is how to effectively control solvent evaporation to make the system stable in the solvent-poor state so as to drive the reaction at a high rate and achieve satisfactory yields. In the past few years, efforts have been made by our groups and others^26^–^30^,^32^,^33^ to achieve this goal. For example, in Leidenfrost droplet synthesis, the solvent can be added back continuously to the levitated droplet to compensate for the solvent loss by evaporation, thus increasing the reaction time in a small controlled-volume reactor.^26^ In thin film deposition synthesis,^29^ small amounts of the reaction mixture are continually added to create a ‘living’ thin film of the precipitated product on an inert surface, again increasing the reaction time for continuous synthesis while retaining the small volume feature needed for acceleration.

**Scheme 1:**
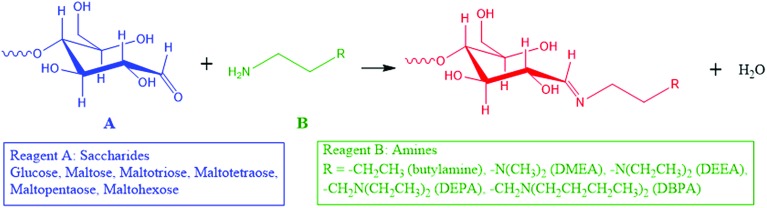
Schiff base reactions between saccharides and amines in this study.

Here, we introduce a reaction strategy for chemical derivatization in ultra-small volumes using a nonvolatile microthin film reactor. Reaction in the microthin film is accelerated while the presence of traces of a nonvolatile solvent in the microthin film increases the reaction time to help achieve very high derivatization yields. The solvent-poor nature of the film means that the equilibrium conditions do not lead to a significant reverse reaction. To characterize the performance of such a microthin film reactor, the Schiff base reaction between a set of primary amines and saccharides (Scheme 1) was studied and the data are compared to bulk reactions run under traditional bulk, neat, and paste conditions. The reasons for this particular choice are (i) Schiff base formation is a reversible reaction with a modest equilibrium constant^34^,^35^ and a reaction rate constant which makes it very difficult to perform chemical derivatization in small volume samples, (ii) identification and quantification of bioactive molecules like saccharides in tissues and single cells is an important task which could lead to deeper understanding of metabolic pathways,^8^,^9^ cell heterogeneity^36^–^40^ and even to the discovery of new drugs^10^–^12^ and (iii) imine formation is an attractive route to efficient ionization of sugars.

## Results and discussion

### Performing reactions in microthin film reactors

**Fig. 1:**
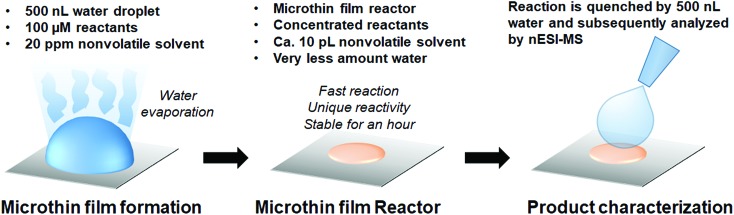
Construction of a microthin film reactor for fast reaction and long reaction times. Typically, a 500 nL aqueous reaction mixture (100 μM saccharide and amine) was dropped on a plate covered with parafilm and left to dry. For amines with high vapor pressure, 20 ppm DMF was added to increase the reaction time. No pH adjustment was made. Accelerated reaction rates and increased extents of reaction could be achieved in the microthin film reactor for an hour. The reaction is allowed to proceed in this reactor for times ranging from several minutes to several hours and subsequently analyzed by nESI-MS. Before MS measurement, the sample from the microthin film was diluted to 500 nL to quench the reaction.

Microthin film reactors were created by allowing evaporation of almost all the water from a 500 nL droplet of an aqueous reaction mixture containing 100 μM nonvolatile amine (*ca.* 20 ppm, 10 pL) and 100 μM saccharide at 25 °C (Fig. 1). Note that very little reaction occurs while most of the 500 nL droplet evaporates. Then, because the aliphatic amines are both nonvolatile and polar, they keep the solvent from evaporating completely so allowing the reaction to proceed in the resulting microthin film reactor for an hour or more. After a chosen reaction time, the reaction is quenched by adding 500 nL water. The quenched solution is subsequently analyzed by nanoelectrospray mass spectrometry (nESI-MS) in the variant that uses an inductive DC voltage.^41^ The apparent acceleration factor (AAF) is used to evaluate reactions in different systems. It is measured as the ratio of ratios of ion abundances of the product [P] relative to the starting material [SM], *viz.* ([P]/[SM]) in the thin film relative to that in bulk after the same reaction time:^28^,^29^
1
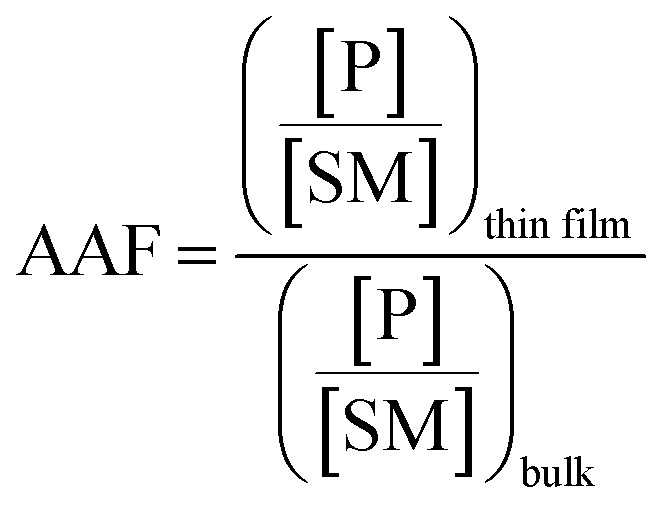



The usefulness of this system for efficient chemical derivatization and chemical analysis is shown below by the highly sensitive mass spectrometric analysis of reducing saccharides in a single *Allium cepa* cell.

### Comparison of microthin film reactions to bulk reactions under different conditions

**Fig. 2:**
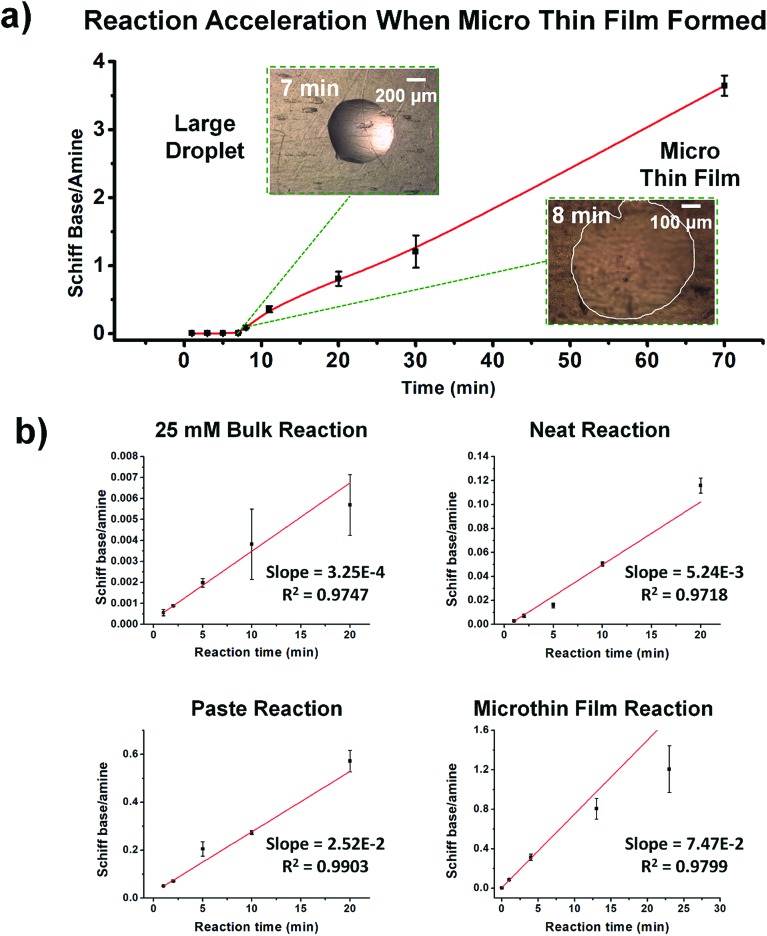
(a) Kinetics of Schiff base reaction in a 500 nL microdroplet under ambient conditions, showing product Schiff base intensity relative to reactant amine intensity. (b) Kinetics curves (fitted based on a second-order rate equation) of bulk reactions between glucose and DBPA under different conditions: saturated bulk reaction (25 mM DBPA and 25 mM glucose), neat reaction (1 mmol glucose and 1 mmol DBPA mixed in a 1.5 mL Eppendorf tube and stirred), paste reaction (1 mmol glucose, 1 mmol DBPA and 100 μL water mixed in a 1.5 mL Eppendorf tube and stirred) and microthin film reaction (100 μM glucose and 100 μM DBPA mixture in a 500 nL droplet and left in an incubator to dry to form a microthin film reactor). Note that the kinetics curves of all bulk reactions fall into the kinetic control region. Since the downward deflection of kinetics curves in the microthin film reactions is due to thermodynamic considerations (see the detailed discussion of the kinetic control region in the ESI‡), only the first four points in the microthin film reaction are fitted. All data were obtained from three replicate experiments.

Fig. 2 shows kinetic data (basis spline fitted) for the reaction between glucose and *N*,*N*-dibutyl-1,3-propanediamine (DBPA) at room temperature (25 °C) from 0 min to 70 min. These mass spectrometric measurements (detailed in ESI, Fig. S1‡) were supplemented by optical microscopy. Note that this reaction was run in the microthin film for up to 24 hours but only data for the first hour have been chosen to examine the reaction kinetics because the microthin thin film dimensions did not change perceptibly during this period. Schiff base reactions are usually second order^34^ so the ratio of product ion intensity/starting material ion intensity, *viz*., ([P]/[SM]), measured from the corresponding ion signals in MS, is plotted against the reaction time to give the kinetics curve. (See ESI,‡ for additional discussion, including the derivation of the equation showing the linear dependence of [P]/[SM] on both the rate constant and the initial concentration.) The kinetics curves of the reaction in the microthin film show two zones. Zone 1 starts with the deposition of the 500 nL droplet and lasts about 7 minutes, while almost all the water evaporates. Microscopy (Fig. S2‡) showed that the reaction system was still in the form of a droplet of a large cross section and MS showed that the extent of reaction was very low during this stage. Zone 2 begins at about 7 minutes, which is when the reaction system was observed to change from a droplet to a microthin film (Fig. S2‡). From the microscopy image in Fig. S2,‡ the cross-sectional shape of the thin film in a typical experiment was constant from 8 min to 70 min. The thin film thickness is not known but it is many thousands of monolayers even after an hour. The kinetics curves in zone 2 are linear, indicating that the reaction occurred under kinetic control over a period of at least 60 min (from 7 min to 70 min). The kinetics curve in zone 2 is used to compare with the kinetics curves of bulk reactions in saturated solution and neat reactants and under “paste” (solvent-poor) conditions. The AAF can be measured from the ratio of the slopes of the kinetics plots in zone 2 *vs.* that of bulk reactions recorded under these different conditions, provided that the system is under kinetic control (see the detailed discussion of the kinetic control region in the ESI‡):2
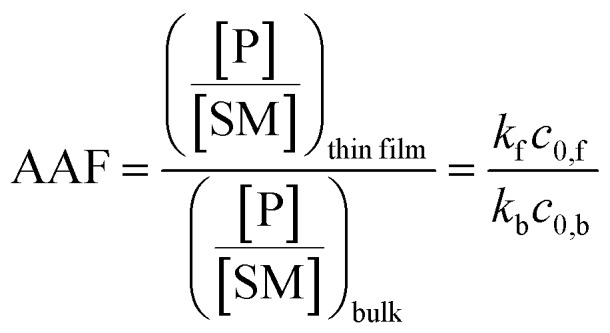
where *k*_f_ and *k*_b_ are the thin film and bulk rate constants and *c*_0,f_ and *c*_0,b_ are the initial concentrations in film and bulk media (see the ESI‡ for derivation and more detailed discussion). From Fig. 1b, among these four reaction conditions, the microthin film reaction appears to have the fastest reaction rate (slope = 7.48 × 10^–2^, *R*^2^ = 0.9799, and RSD = 5.6%), which is 3.0 (RSD = 5.6%) times faster than the paste reaction (slope = 2.52 × 10^–2^, *R*^2^ = 0.9903, and RSD = 3.6%), 14.2 times (RSD = 3.6%) faster than the neat reaction (slope = 5.24 × 10^–3^, *R*^2^ = 0.9718, and RSD = 3.2%) and 230 times (RSD = 14.5%) faster than the bulk reaction at 25 mM with saturated DBPA (slope = 3.25 × 10^–4^, *R*^2^ = 0.9747, and RSD = 14.5%). As the solubility of DBPA in water is not good, 25 mM is the maximum concentration possible for the homogenous bulk reaction. The significant difference in the reaction rate between the microthin film reaction and the bulk reaction with saturated DBPA suggests a heterogeneous reaction mechanism of the fast reaction in the microthin film. It is notable that the heterogeneous reaction mechanism is also used to describe the reaction under neat and paste conditions.^14^,^15^ In contrast to the neat and paste reactions, in the microthin film reaction the reaction mixture is derived from solvent evaporation of a homogeneous droplet, ensuring that reactants in the heterogeneous microthin film are well mixed. Evaporation enhances mass transfer efficiency through preconcentration. This accounts for an increase in the reaction rate in the microthin film reactor by a factor of 2 over the paste reaction and almost 10 over the neat reaction. According to eqn (2), the AAF in different media is determined by the ratio of the intrinsic reaction rate constants and by the initial reagent concentration. In the microthin film, neat and paste reactions, the reactants are highly concentrated and over-saturated, and therefore the ratio of concentrations is *ca.* 1 and under these circumstances, the AAF actually reflects the difference in intrinsic rate constants.

### Solvent effect on microthin film reactions

Further information on the role of solvation in reactions in microthin films comes from experiments using solvents of different vapor pressures and polarities to construct the microthin film. In Table 1, using nonvolatile reagents, we found that solvents of higher vapor pressure facilitate microthin film reactions, probably because of the increased concentration when less solvent remains in the microthin film reactor at 25 °C. In detail, water, isopropanol and methanol, with increasing vapor pressures of 23.7 torr, 45.4 torr and 127 torr, show increasing conversion ratios after a 20 min reaction of 55%, 78% and 87%, respectively. An extreme case is that of DMF, a nonvolatile solvent, which showed a conversion ratio of less than 1%. Besides vapor pressure, polar solvents show significantly higher conversion ratios than nonpolar solvents. For example, although cyclohexane and hexane have standard vapor pressures of 96.9 torr and 153 torr, their conversion ratios are only 5% and 9%. This is consistent with more effective solvation of the amino group of DBPA and the hydroxyl groups of glucose by the polar solvent, making it easier for the amino group to attack glucose.

**Table 1:** Conversion ratio ([P]/([SM] + [P])) for reactions between DBPA and glucose using different solvents^*a*^

Solvent name	Polarity	25 °C vapor pressure (torr)	Conversion ratio (%)
Methanol	Polar	127	86 ± 2
Isopropanol	Polar	45.4	75 ± 4
Water	Polar	23.7	51.2 ± 4
DMF	Polar	3.87	<0.1
CHEX	Nonpolar	96.9	6 ± 3
Hex	Nonpolar	153	9 ± 1

^*a*^Total reaction time was 20 min (8 min for droplet evaporation and 12 min in the microthin film form); conversion ratio was reported here to emphasize yield differences between different systems. Data were obtained from three replicate experiments.

### Temperature effects on microthin film reactions

**Fig. 3:**
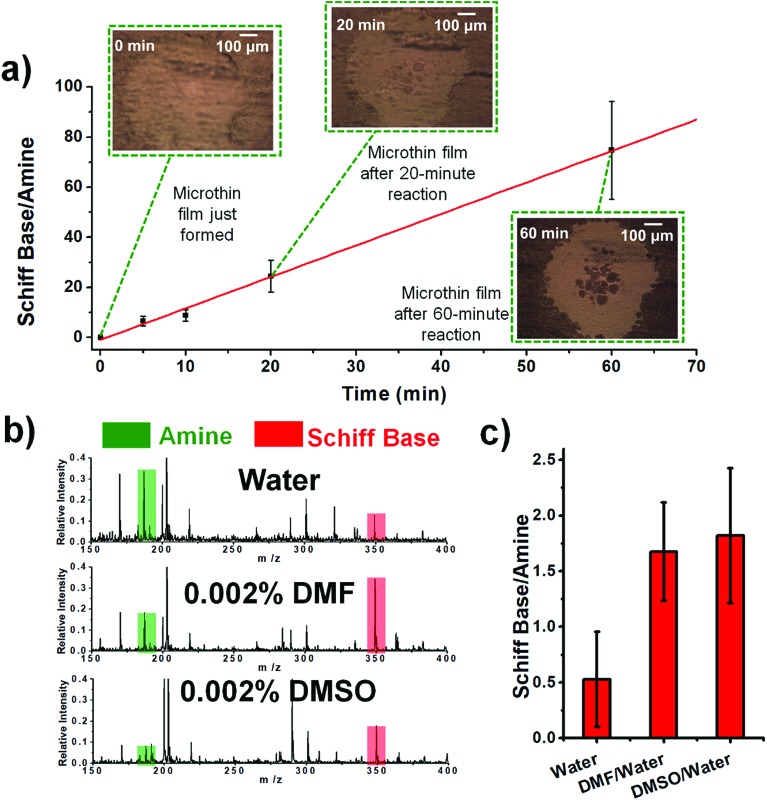
(a) Kinetics curve for the reaction between aqueous DBPA and glucose in 65 °C. The droplet was first held at 25 °C for 8 min to ensure that the microthin film had formed and then held at 65 °C for the reaction. (b) Schiff base reaction between 100 μM glucose and 10 μM DBPA in the microthin film for 10 min with water, 0.002% DMF in water and 0.002% DMSO in water as the solvent. (c) [P]/[SM] after a 10 min reaction in different solvents. All data were obtained from three replicate experiments.

The temperature was also examined as a variable affecting thin film reactivity. When the reaction between DBPA and glucose was performed in a microthin film at 65 °C, the kinetics curve (Fig. 3a) again showed very good linearity between the reaction time and ratio, [P]/[SM]. However, the slope was 1.23 min^–1^ (*R*^2^ = 0.9973, RSD = 3.1%), which is 24.4 times that for the reaction at 25 °C. A common explanation of the accelerated reaction rate is the increase of the intrinsic rate constant at the higher reaction temperature. However, we also found that less solvent is left in the microthin film reactor at a higher temperature (Fig. S4‡) and suggest that this also contributes to the observed rate increase. The advantage of increased temperature is clear but at about 60 minutes rupture of the thin film was observed so that it no longer had a single continuous surface. The reaction between glucose and amines of different vapor pressures at 65 °C showed values of [P]/[SM] after 20 min of reaction that correlated negatively with the amine vapor pressure (Fig. S5‡). Several other observations suggested that the nonvolatile primary amine plays a key role in stabilizing the microthin film. Use of large vapor pressure amines or decreasing the amine amount made the thin film extremely fragile. This problem could be solved by using mixed solvents to generate the thin film. When using just 0.002% (v/v) DMF or DMSO in the DBPA/glucose reaction, [P]/[SM] increased from 0.5 (water) to 1.7 and 1.8, respectively (Fig. 3b and c). Besides amine vapor pressure, amine basicity may also influence the reaction. For the nonvolatile amines DMEA, DEEA, DEPA and DBPA, p*K*_a_1__ is 6.6, 7.0, 7.7 and 8.0 (data source: ; https://www.chemicalize.com), respectively, which correlates positively with the [P]/[SM] value of 9.0, 15.9, 18.7 and 21.3. However, basicity is a secondary factor relative to vapor pressure. For example, although butylamine, a volatile amine, has a p*K*_a_ of 10.2 the [P]/[SM] value is only 0.34.

### Reaction yields in microthin film reactions

Besides reaction acceleration, an even more important characteristic of microthin film reactors, which could have broad application, is the significant yield enhancement available. High yields depend on being able to run reactions for appropriately long times, which we have seen is readily achieved in microthin film reactors. They also require large equilibrium constants, especially when the substrate is present in low concentration. The Schiff base reaction is normally not suitable for chemical derivatization because it is a reversible reaction with a small equilibrium constant under standard conditions. For example, the reaction of protonated *p*-chlorobenzaldehyde and aniline hydrochloride to yield protonated *N-p*-chlorobenzylideneaniline has an equilibrium constant of only 0.08.^34^ When using 100 μM DBPA to derivatize 100 μM of a saccharide (glucose, maltose, maltotriose, maltotetraose, maltopentaose and maltohexaose) in bulk solution at 65 °C, the [P]/[SM] values of all six reactions after 24 hours were less than 0.005 (conversion ratio < 0.5%) (Table 2). However, using the microthin film reactor for derivatization, the [P]/[SM] value was 2.01 to 22.1 (conversion ratio 66.8% to 95.7%), *viz.* 500 to 6000 times the bulk reaction value. Notably, the reaction takes only 20 minutes compared to 24 hours for completion in bulk. The increase in the reaction yield is the result of three features of the microthin film reactor strategy: (i) the concentrations of reactants can be as high as that in a saturated solution, (ii) the fraction of molecules near the surface is maximized, and (iii) the open reaction system promotes escape of water produced in the reaction. This latter factor increases the reaction conversion ratio and it also accelerates the reaction according to the rate equation of reversible reactions (see ESI eqn (9)–(15) for derivations and extended discussion‡).

**Table 2:** Conversion ratio (CR) for reactions between DBPA and different saccharides in a traditional bulk reactor and a microthin film reactor. Both reactions were at 65 °C and data were taken at 24 hours for the bulk reaction and 20 minutes for the microthin film reaction

Saccharides	CR (%) in bulk (24 hours)	CR (%) in microthin films (20 minutes)
Glucose	0.5 ± 0.3	95.7 ± 0.4
Maltose	0.5 ± 0.2	90.2 ± 1.2
Maltotriose	0.4 ± 0.2	86.3 ± 1
Maltotetraose	0.5 ± 0.2	72.3 ± 2.6
Maltopentaose	0.4 ± 0.2	70.4 ± 5.8
Maltohexose	0.4 ± 0.1	64.9 ± 10.1

### Utilization of thin film reactions for derivatization of saccharides in single cells

**Fig. 4:**
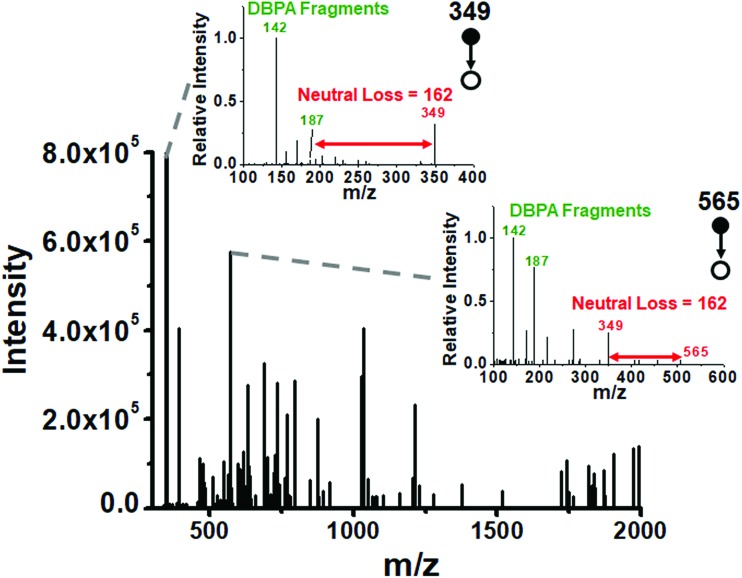
Extracted *m*/*z* values of two ions which undergo a neutral loss of 162 (red arrows) and DBPA derived fragments (*m*/*z* 142 and 187) in MS^2^ product ion spectra (inset) after single cell fluid derivatization. There are 125 peaks in the mass spectrum which have structures derived from DBPA and a saccharide.

We utilized the microthin film reactor to perform Schiff base derivatization of the saccharides from the intracellular fluid of a single cell. The extract of an individual *Allium cepa* cell (*ca.* 1 nL) was taken up in a nESI emitter tip using a 3D micromanipulator. The intracellular glucose concentrations reported for some animal cells are 2 to 50 mM 42 while they are 50 mM to 125 mM 43 in *Allium cepa* bulbs, depending on storage time. Using a value of 100 mM as the glucose concentration, we estimate the amount of glucose in 1 nL of single cell extract as 18 ng (0.1 nmol) with smaller amounts of other sugars. To ensure good derivatization efficiency of all reducing saccharides in the cell, the tip of the emitter was fractured and the small sample-containing end section was immersed in a 500 nL droplet containing 10 mM DBPA (50 times excess over glucose) to perform the microthin film Schiff base derivatization at 65 °C for 20 minutes (see the schematic diagram in Fig. S7‡). The derivatized saccharides showed typical fragment ions in their product ion MS^2^ spectra (Fig. 4). For example, the fragment ions *m*/*z* 142 and 187 are characteristic imine derived fragments and the neutral loss of 162 indicates the hexose unit. We used a data dependent scan strategy to record the collision-induced dissociation of the 1050 most abundant peaks in the full scan MS; 125 of these spectra showed typical sugar neutral losses of 162 and DBPA derived fragment ions at 142 and 187. We successfully identified 29 reducing saccharides from these 125 spectra (Table S2‡), demonstrating the microthin film reactor to be a simple yet powerful tool for the rapid derivatization and identification of low nanogram level analytes in single cells.

## Conclusion

The microthin film reactor utilized in this study can be contrasted with that used in an earlier study^29^ in which a very large acceleration factor together with continuous reagent addition was used to scale up product yields while the thin film of the reaction solution was maintained by continuous product precipitation. Product removal by precipitation there avoided concerns regarding rate decrease by the back reaction in cases where the equilibrium constant is not large. However, even if the equilibrium constant is small, as represented by this study, reaction yields can be high enough to allow successful derivatization on a picomole to nanomole scale if measures are taken to increase reaction times while working with very small amounts of reagent.

## Experimental section

### Materials

All saccharides (glucose, maltose, maltotriose, maltotetraose, maltopentaose and maltohexaose) were purchased from Beijing Bio-dee Biotechnology Co. Ltd. All amines (butylamine, *N*,*N*-dimethyl-1,2-ethylenediamine (DMEA), *N*,*N*-diethyl-1,2-diaminoethane (DEEA), *N*,*N*-diethyl-1,3-propanediamine (DEPA) and *N*,*N*-dibutyl-1,3-propanediamine (DBPA)), DMF and DMSO were purchased from Beijing Hwrk Chemical Co. Ltd. The white *Allium cepa* bulb was purchased from a local market. All stock solutions were stored at 4 °C before use.

### Reactions

Unless otherwise noted the concentration of reactants for Schiff base reactions was 100 μM. For bulk reactions, a 200 μL reaction mixture was placed in an Eppendorf tube in an incubator to control the reaction time and temperature. For microthin film reactions, parafilm was attached to a glass slide to form a hydrophobic surface. Then 500 nL of the reaction mixture was cast on the parafilm to form the reaction solution. The plate was placed in an incubator to control the temperature at either 25 °C or 65 °C. For all microthin film experiments, 500 nL reaction solution containing all reagents is left to dry at 25 °C for 7 minutes to form the microthin film reactors.

### Single cell derivatization

A nanoelectrospray emitter was controlled by using a precise moving stage to extract ∼1 nL of fluid from a single *Allium cepa* cell. The end of the emitter tip was then cut off and inserted into a 500 nL droplet (DBPA 10 mM) to allow Schiff base reaction once the droplet was placed in an incubator for 10 min in 65 °C. This single sample was used for MS/MS based sugar and its modification identification.

### Mass spectrometry

Except for the single cell analysis, all other experiments were carried out using a Thermo LTQ mass spectrometer (Thermo Scientific, San Jose CA). The instrumental parameters were as follows: capillary temperature = 275 °C and max injection time = 200 ms. The single cell amount analysis was carried out using a Thermo QE-Orbitrap mass spectrometer (Thermo Scientific, San Jose CA). The capillary temperature was 320 °C. The analytical method was full MS, followed by data dependent MS^2^. The method duration was 5 min. Full MS: resolution = 70 000, AGC = 3 × 10^6^, max injection time = 100 ms, and scan range = 134 to 2000. The ddMS^2^ settings: resolution = 35 000, AGC = 1 × 10^5^, ion injection time = 100 ms, loop count = 10, NCE = 30, underfill ratio = 0.1%, and dynamic exclusion = 300 s.

### Microscopy

A metallurgical microscope (YX20L20, Dayueweijia Science and Technology Co. Ltd., Beijing) was used to examine the microthin film. Photos of the microthin film were taken under the following conditions: reactant concentrations were 10 mM; droplet volume was 500 nL.

## Conflicts of interest

The authors declare no conflicts of interest.

## Supplementary Material

Supplementary information
