# Supplementary material for: High yield accelerated reactions in nonvolatile microthin films: chemical derivatization for analysis of single-cell intracellular fluid
Source: Chem Sci. 2018 Aug 21;9(40):7779–86. doi: 10.1039/c8sc03382j (PMC6195031; doi:10.1039/c8sc03382j)
Supplement: Supplementary file 1 [file SC-009-C8SC03382J-s001.pdf]

Electronic Supplementary Material (ESI) for Chemical Science.
This journal is © The Royal Society of Chemistry 2018

Supporting Information

High Yield Accelerated Reactions in Nonvolatile Microthin Films: Chemical Derivatization for Analysis of Single-Cell Intracellular Fluid

Zhenwei Wei, Xiaochao Zhang, Jinyu Wang, Sichun Zhang, Xinrong Zhang and R. Graham Cooks**

Table of Contents

Additional Discussion 2

Kinetics of Second Order Irreversible Reactions 2

Kinetics of Second Order Reversible Reactions 3

Figures and Tables 6

Figure S1. Typical mass spectra for Schiff base reaction between DBPA and glucose in the microthin film reactor. The reaction temperature was 25 °C 6

Figure S2. Typical micrographs of the microthin film reactor at different reaction times. The reaction temperature was 25 °C 7

Figure S3. Typical mass spectra for Schiff base reaction between DBPA and glucose in microthin film reactor. The reaction temperature was 65 °C 7

Figure S4. Typical micrographs of the microthin film reactor showing shape at different reaction times. The reaction temperature was 65 °C 8

Table S1. Physical properties of different amine reagents used in Schiff base reactions 8

Figure S5. Schiff base reaction between glucose and different primary amines in microthin film at 65 °C for 20 minutes. Intensities of peaks due to Schiff base product and glucose peak were used to calculate [P]/[SM] for the comparison....................9

Figure S6. Reaction of glucose and DBPA in 25 °C microthin film. [P]/[SM] at six time points as recorded by nESI. 9

Figure S7. Procedure to perform chemical derivatization of individual cell extract in microthin film reactor. 10

Table S2. Identification of 29 reducing saccharides in a single Allium cepa cell and their structural modifications relative to glucose. 10

Reference 13

Additional Discussion

Kinetics of Second Order Irreversible Reactions

Consider the Schiff base reaction below:

$$SM_1 + SM_2 \rightarrow P + H_2O$$

Considering reaction with equal amounts of reactants, assume that the concentration of reactants SM_1 and SM_2 are both c_0 at the start. At any reaction time $t > 0$, $[SM_1] = [SM_2] = c$, and $[P] = c_0 - c$, the reaction rate is r. According to rate equation we can get:

$$r = -\frac{dc}{dt} = kc^2 \tag{1}$$

Integrating eq. (1) we get:

$$\frac{1}{c} - \frac{1}{c_0} = kt \tag{2}$$

Note that the product concentration $[P] = c_0 - c$, and starting material concentration $[SM_1] = [SM_2] = c$, so eq. (2) can be rewritten as:

$$\frac{[P]}{[SM]} = c_0 kt \tag{3}$$

Therefore, [P]/[SM] is plotted against reaction time t to create the kinetics curve. Notably, the slope of the kinetics curve is a function of c_0. If the slopes of two reaction systems are compared (their ratio representing the **apparent acceleration factor**),[1] we find a dependence on both the ratio of concentrations and the rate constants (the latter being the **rate acceleration factor**). For example, when comparing system 1 and system 2, we can get:

$$AAF = \frac{(\frac{[P]}{[SM]}/t)_{system\ 1}}{(\frac{[P]}{[SM]}/t)_{system\ 2}} = \frac{slope_1}{slope_2} = \frac{c_{0,1}}{c_{0,2}} \times \frac{k_1}{k_2} \tag{4}$$

When performing analysis by mass spectrometry, concentrations are estimated

using ion abundances. The ionization efficiency of the amine in a Schiff base reaction is significantly higher than that for a saccharide but similar to that of the Schiff base product. Therefore, the concentration ratio [P]/[SM] can be approximated by the ratio of ion abundances (peak intensities of the Schiff base product vs. the amine). The conversion ratio is measured by Schiff base ion intensity/(Schiff base intensity + amine ion intensity). This again assumes, as is common, that ionization efficiency similarities allow ion abundances to stand in for concentrations, as discussed, for example, in a previous publication.[2]

Kinetics of Second Order Reversible Reactions

There is no absolutely irreversible reaction. Every reaction given a finite temperature, will reach equilibrium if the reaction time is sufficient. Reaction equilibrium is reached when the forward reaction rate is equal to the backward reaction rate. Consider a second order reversible reaction generating a larger molecule (P) with the loss of a small molecule (W):

$$SM_1 + SM_2 \rightleftarrows P + W$$

When equilibrium is reached, we can get:

$$k_f[SM_1][SM_2] = k_b[P][W] \tag{5}$$

In equation 5, k_f and k_b are the rate constants for the forward and backward reactions, respectively. Rearranging eq. 5 we can get the well-known relationship between k_f, k_b and equilibrium constant K:

$$\frac{k_f}{k_b} = \frac{[P][W]}{[SM_1][SM_2]} = K \tag{6}$$

Consider the situation that all reactants have equal initial concentrations, c_0. At time t, $[SM_1] = [SM_2] = c$, $[P] = [W] = c_0 - c$. Considering the backward reaction, the reaction rate equation can be rewritten as follows:

$$r = -\frac{dc}{dt} = k_f[SM_1][SM_2] - \frac{k_f}{K}[P][W] = k_f(c^2 - \frac{1}{K}(c_0 - c)[W]) \tag{7}$$

From equation 7, if the equilibrium constant K is large enough, or $[P] = c_0 - c = [W]$ is small enough, we will get:

$$c^2 \gg \frac{1}{K}(c_0 - c)[W] \tag{8}$$

Under these circumstances The rate equation will change to the form of the irreversible

reaction shown in eq. 1. As Schiff base reactions are typically reversible reactions with K values about 10, the above situation would only occur when $[P] = c_0 - c$ or $[W]$ is very small. Note the two alternatives which occur with introduction of the implicit qualification that $[P]$ and $[W]$ might not be identical (considering that the small product W might evaporate from the reaction mixture). In the first case, $[P] = c_0 - c$ is very small. This indicates that we can use the irreversible rate equation to describe reaction kinetics when the conversion of reagents to products is small. For example, Figure S6 shows the Schiff base reaction in 25 °C microthin film for 1440 minutes. The first five 5 points were acquired at reaction times of less than 70 minutes and [P]/[SM] is less than 3. In this region, the extent of reaction is not high enough to generate large amounts of product so the backward reaction is still negligible. This explains why the first 5 points show a very good linear relationship in Figure S6 but the sixth point shows a downward deflection from the line. For the sake of discussion, we can call the region that obeys the irreversible rate equation the **kinetics-controlled region** and the region that shows deflection from the irreversible rate equation curve the **thermodynamics-controlled region**. Obviously, the reaction rate in the kinetics-controlled region is the maximum reaction rate. Given these definitions, we discuss the second case, that in which $[W]$ is very small. This occurs in the 65 °C microthin film reaction, in which the thinner film and the higher temperature contribute enormously to water escaping from the microthin film reactor. Comparing Figure 3a and Figure S6, [P]/[SM] plotted against reaction time in the 25 °C microthin film follows a linear relationship even when [P]/[SM] is more than 70 (conversion > 98.5%) while we see downward deflection in the 65 °C microthin film when [P]/[SM] is less than 3 (conversion < 75%). This result indicates that the ease of water escaping in the microthin film not only enhances the reaction yield, but also increases the period of kinetics-control of the reaction.

For reaction in a microthin film, a reasonable assumption is that the rate of small molecules escaping from the system is faster than the reaction rate (otherwise the water generated by reaction could lead to an increase in the volume increase of the thin film).This assumption helps us to solve equation 7. Let's assume that [W] = w, which is constant for the same microthin film and environment. We can rewrite equation 7 as follows:

$$-\frac{dc}{dt} = k_f\left(c^2 - \frac{1}{K}(c_0 - c)[W]\right) = k_f\left(c^2 + \frac{w}{K}c - \frac{w}{K}c_0\right)$$

$$= k_f\left(c^2 + \frac{w}{K}c + \frac{w^2}{4K^2} - \frac{w^2}{4K^2} - \frac{w}{K}c_0\right) = k_f\left((c + \frac{w}{2K})^2 - (\frac{w^2}{4K^2} + \frac{w}{K}c_0)\right) \quad (9)$$

Rearranging this equation we get:

$$k_f \cdot dt = -\frac{1}{(c + \frac{w}{2K})^2 - (\frac{w^2}{4K^2} + \frac{w}{K}c_0)} \cdot dc$$

$$=\frac{1}{2\sqrt{\frac{w^2}{4K^2}+\frac{wc_0}{K}}}\left(\frac{1}{c+\frac{w}{2K}+\sqrt{\frac{w^2}{4K^2}+\frac{wc_0}{K}}}-\frac{1}{c+\frac{w}{2K}-\sqrt{\frac{w^2}{4K^2}+\frac{wc_0}{K}}}\right)\cdot dc \tag{10}$$

Integrating equation 10 we can get:

$$ln\frac{c+\frac{w}{2K}+\sqrt{\frac{w^2}{4K^2}+\frac{wc_0}{K}}}{c+\frac{w}{2K}-\sqrt{\frac{w^2}{4K^2}+\frac{wc_0}{K}}}=k_f\sqrt{\frac{w^2}{K^2}+\frac{4wc_0}{K}}\cdot t+ln\frac{c_0+\frac{w}{2K}+\sqrt{\frac{w^2}{4K^2}+\frac{wc_0}{K}}}{c_0+\frac{w}{2K}-\sqrt{\frac{w^2}{4K^2}+\frac{wc_0}{K}}} \tag{11}$$

Assuming that when reaction reaches equilibrium, $[SM_1] = [SM_2] = c_e$, we can solve for the equilibrium concentration c_e from the following equation:

$$K=\frac{[P][W]}{[SM_1][SM_2]}=\frac{(c_0-c_e)w}{c_e^2} \tag{12}$$

$$c_e=-\frac{w}{2K}+\sqrt{\frac{w^2}{4K^2}+\frac{wc_0}{K}} \tag{13}$$

Combining equations 11 and 13, equation 11 can be rewritten as:

$$ln\frac{c+c_e+\frac{w}{K}}{c-c_e}=k_f\sqrt{\frac{w^2}{K^2}+\frac{4wc_0}{K}}\cdot t+ln\frac{c_0+c_e+\frac{w}{K}}{c_0-c_e} \tag{14}$$

Setting $2c_e+\frac{w}{K}=\sqrt{\frac{w^2}{K^2}+\frac{4wc_0}{K}}=a$, eq.13 can be rewritten as:

$$ln(1+\frac{a}{c-c_e})=ak_f\cdot t+ln(1+\frac{a}{c_0-c_e}) \tag{15}$$

From equation 15, we find that a is related to reaction thermodynamics only and k_f is only related to the reaction kinetics. These two parameters express the thermodynamic control and the kinetic control of the reaction rate.

It is notable that AAF is a measure of both extent of reaction and the intrinsic rate acceleration factor (k_1/k_2). To calculate AAF, the apparent acceleration factor, one just needs to compare the ratio of [P]/[SM] of different reactions (([P1]/[SM1])/([P2]/[SM2])) at fixed reaction time. For reversible second order reaction, if AAF is calculated in the kinetic control region, AAF is $k_f c_{o,f}/ k_b c_{o,b}$; if calculated in both kinetic and thermodynamic control region (at high reaction conversion ratio), AAF just tells the average reaction rate ratio for different reactions. Although AAF is just crude comparison for reaction rates in different systems, but it is easy to get and could be used to evaluate the reaction rate of different systems.

Figures and Tables

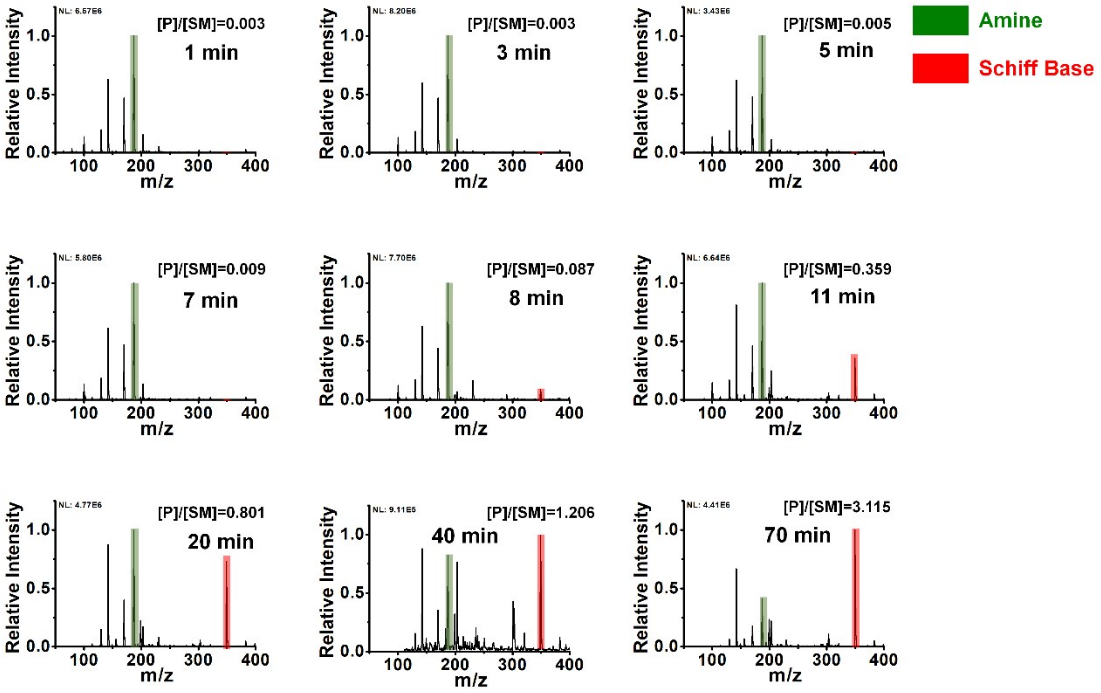

Figure S1. Typical mass spectra for Schiff base reaction between DBPA and glucose in the microthin film reactor. The reaction temperature was 25 °C**.**

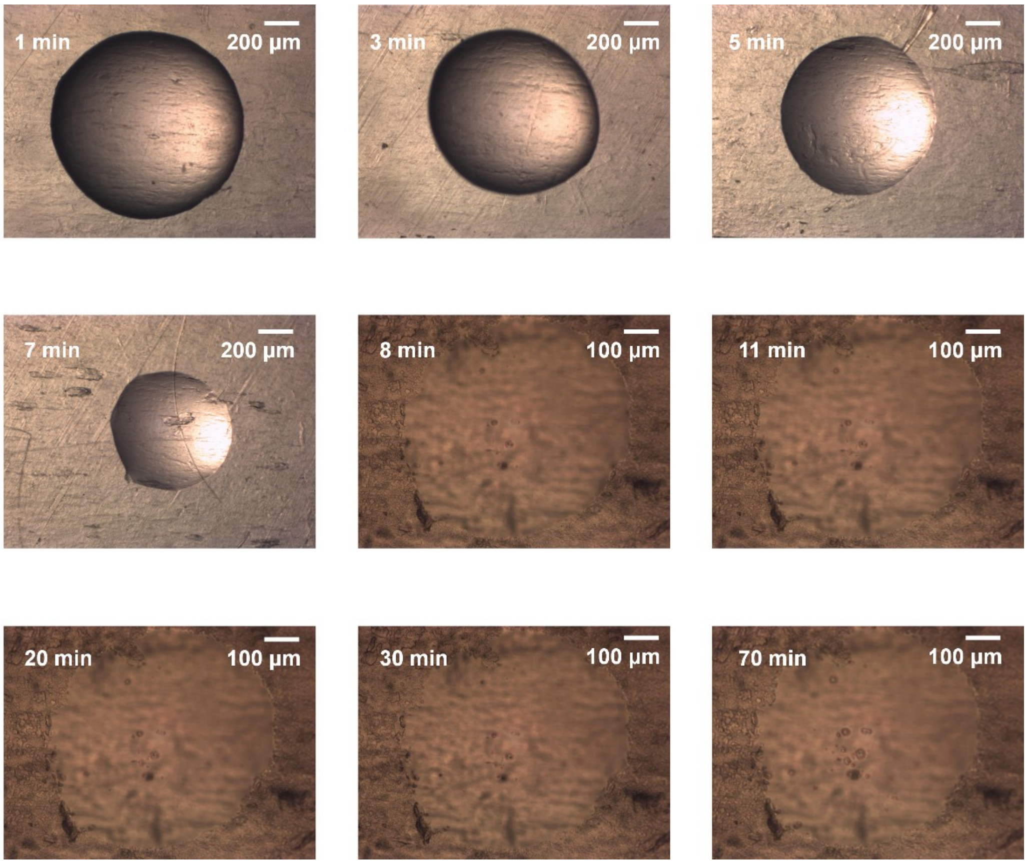

Figure S2. Typical micrographs of the microthin film reactor at different reaction times. The reaction temperature was 25 °C.

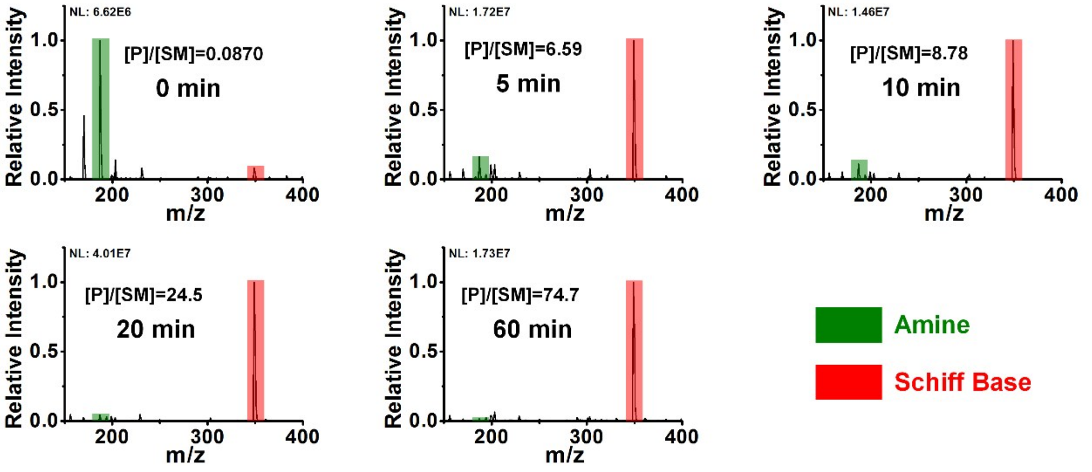

Figure S3. Typical mass spectra for Schiff base reaction between DBPA and glucose in microthin film reactor. The reaction temperature was 65 °C.

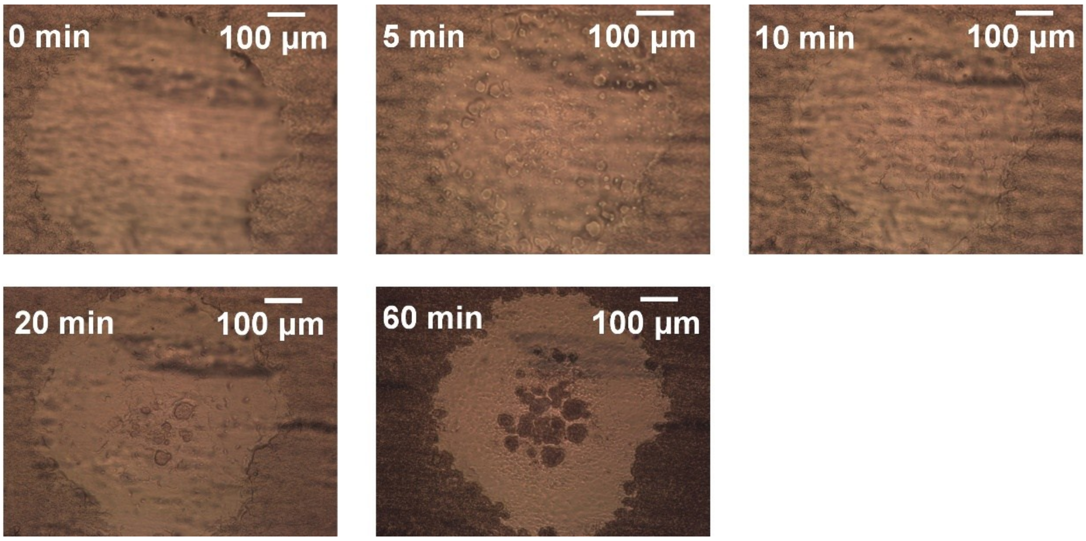

Figure S4. Typical micrographs of the microthin film reactor showing shape at different reaction times. The reaction temperature was 65 °C.

Table S1. Physical properties of different amine reagents used in Schiff base reactions**.**

Amine	Structure	B.P./°C	Vapour Pressure/mm Hg (25°C)
Butylamine	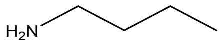	78	68
N,*N*-Dimethyl-1,2-ethylenediamine (DMEA)	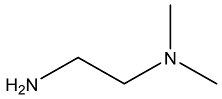	93.5	49.3
N,*N*-Diethyl-1,2-diaminoethane (DEEA)	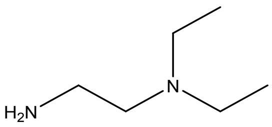	143	5.56
N,*N*-Diethyl-1,3-propanediamine (DEPA)	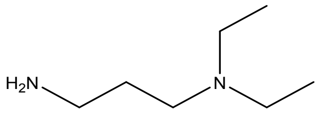	170	1.45

N,*N*-Dibutyl-1,3-propanediamine (DBPA)	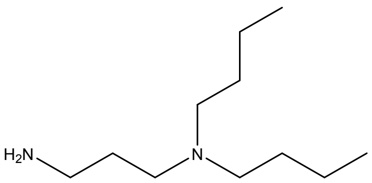	205	0.256

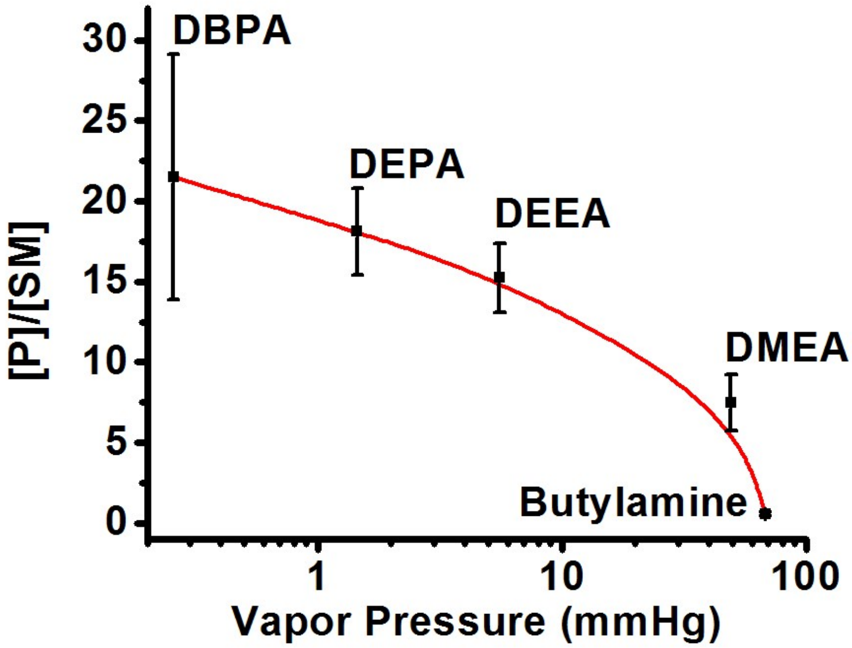

Figure S5. Schiff base reaction between glucose and different primary amines in microthin film at 65 °C for 20 minutes. Intensities of peaks due to Schiff base product and glucose peak were used to calculate [P]/[SM] for the comparison.

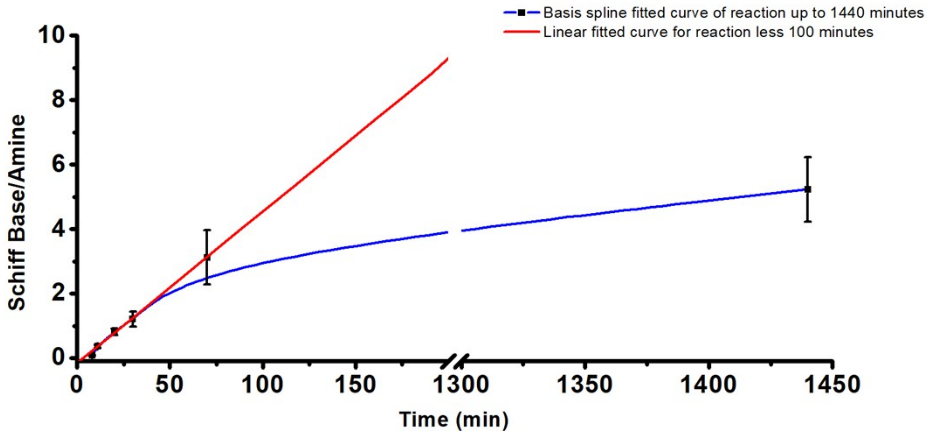

Figure S6. Reaction of glucose and DBPA in 25 °C microthin film. [P]/[SM] at six time points as recorded by nESI. The Schiff base reaction is a reversible reaction,

however, the first five points show a good linear relationship following the second order irreversible reaction rate equation (red curve). After 70 minutes, however, as the reaction approaches equilibrium and the backward reaction rate cannot be neglected. This cause the downward deflection of the linear rate equation curve (blue line).

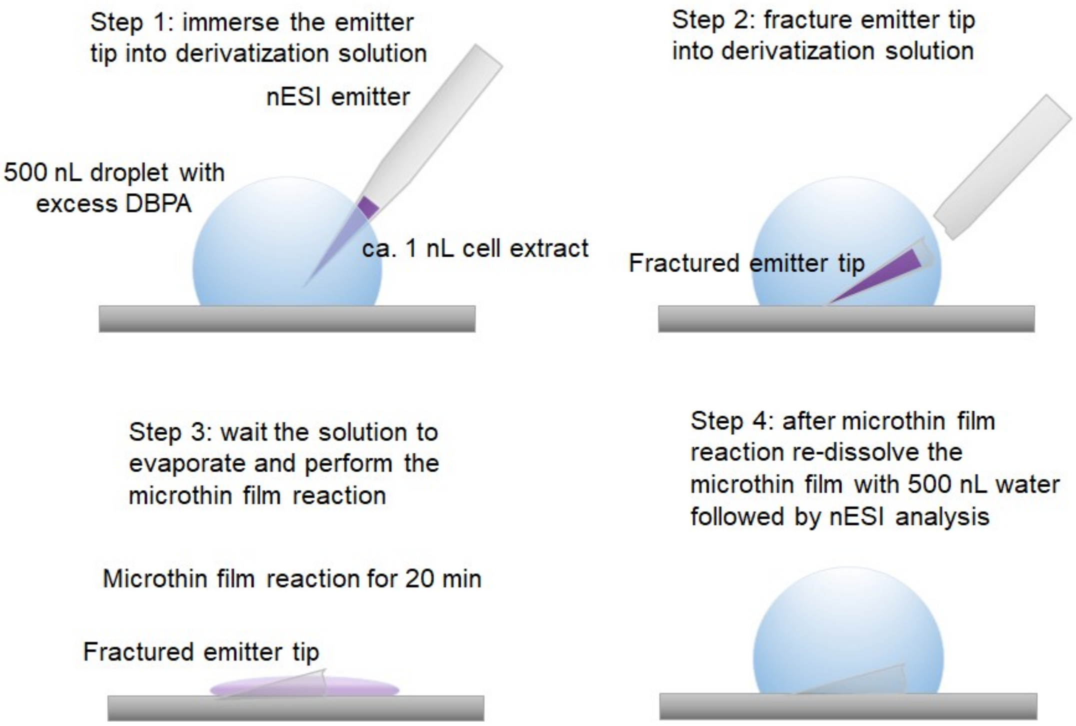

Figure S7. Procedure to perform chemical derivatization of individual cell extract in microthin film reactor.

Table S2. Identification of 29 reducing saccharides in a single Allium cepa cell and their structural modifications relative to glucose.

m/z	Possible Molecular Formula	# of Saccharide Unit(s)	Structural Modification	Mass Deviation (‰)
349.30	$C_6H_{12}O_6$	1	N.A.	0.10
363.09	$C_7H_{14}O_6$	1	CH_2	-0.52

393.28	$C_7H_{12}O_8$	1	CO_2	-0.52
394.3	$C_7H_{15}NO_7$	1	O,CH_2NH	-0.04
395.06	$C_8H_{18}O_7$	1	C_2H_4， H_2O	-0.38
419.29	$C_{10}H_{18}O_7$	1	$COCH_2$， C_2H_4	-0.03
511.46	$C_{12}H_{22}O_{11}$	2	N.A.	0.27
527.46	$C_{12}H_{22}O_{12}$	2	O	0.36
529.47	$C_{12}H_{24}O_{12}$	2	H_2O	0.21
537.10	$C_{14}H_{24}O_{11}$	2	C_2H_2	-0.19
557.47	$C_{14}H_{28}O_{12}$	2	C_2H_4， H_2O	0.36
565.45	$C_{16}H_{28}O_{11}$	2	C_4H_6	0.14
573.50	$C_{13}H_{28}N_2O_{12}$	2	O,CH_2NH, NH_3	0.24
574.46	$C_{13}H_{27}NO_{13}$	2	O,CH_2NH, H_2O	0.24
599.48	$C_{14}H_{22}O_{15}$	2	$CO_2 \times 2$	0.36
615.44	$C_{17}H_{35}N_2O_{11}$	2	C_5H_{10}， $NH_3 \times 2$	-0.15
637.33	$C_{18}H_{28}O_{14}$	2	$COCH_2 \times 3$	-0.10
701.51	$C_{20}H_{36}O_{16}$	3	C_2H_4	-0.16
727.33	$C_{22}H_{38}O_{16}$	3	C_4H_6	-0.13
761.54	$C_{20}H_{32}O_{20}$	3	$CO_2 \times 2$	0.29
878.56	$C_{25}H_{43}NO_{22}$	4	CONH	0.09
1204.52	$C_{37}H_{65}NO_{32}$	6	O,CH_2NH	-0.04

1213.69	$C_{40}H_{68}O_{31}$	6	C_4H_6	0.10
1375.73	$C_{46}H_{78}O_{36}$	7	C_4H_6	0.06
1518.79	$C_{48}H_{87}NO_{42}$	8	H_2O，NH_3	0.11
1720.64	$C_{57}H_{101}NO_{47}$	9	O，C_3H_6，NH_3	-0.05
1742.13	$C_{61}H_{104}O_{46}$	9	C_7H_{12}	0.14
1825.72	$C_{60}H_{104}O_{52}$	10	H_2O	-0.02
1836.63	$C_{61}H_{105}NO_{51}$	10	CH_2NH	-0.11

Reference

[1] R. M. Bain, C. J. Pulliam, F. Thery, et al., *Angew. Chem. Int. Edit.* **2016**, *55*, 10478-10482.

[2] Z. W. Wei, M. Wleklinski, C. Ferreira, et al., *Angew. Chem. Int. Edit.* **2017**, *56*, 9386-9390.
